# Global vaccine action plan lessons learned I: Recommendations for the next decade

**DOI:** 10.1016/j.vaccine.2020.05.003

**Published:** 2020-07-14

**Authors:** Noni MacDonald, Ezzeddine Mohsni, Yagob Al-Mazrou, Jon Kim Andrus, Narendra Arora, Susan Elden, Marie-Yvette Madrid, Rebecca Martin, Amani Mahmoud Mustafa, Helen Rees, David Salisbury, Qinjian Zhao, Ian Jones, Christoph A. Steffen, Joachim Hombach, Katherine L. O'Brien, Alejandro Cravioto

**Affiliations:** a SAGE Decade of Vaccines Working Group; b Professor of Paediatrics, Dalhousie University, IWK Health Centre, Canada. Chair of the SAGE Decade of Vaccines Working Group; c Senior Technical Adviser in Global Health Development/Eastern Mediterranean Public Health Network; d Secretary General - Health Services Council of the Kingdom of Saudi Arabia, Saudi Arabia; e Adjunct Professor and Director, Division of Vaccines and Immunization, Center for Global Health, University of Colorado, USA; f Executive director, International Clinical Epidemiology Network, India; g Health Adviser, Department for International Development, London, UK; h Independent Consultant, Geneva, Switzerland; i Director of the Center for Global Health, US CDC, USA; j Project Manager, Sudan Public Health Training Initiative, Carter Center, Sudan; k Executive Director, Wits Reproductive Health and HIV Institute, Personal Professor, Ob/Gyn Codirector, African Leadership in Vaccinology Excellence, University of Witwatersrand, South Africa; k Associate Fellow, Centre on Global Health Security, Chatham House, London, UK; m State Key Laboratory of Molecular Vaccinology and Molecular Diagnostics, National Institute of Diagnostics and Vaccine Development in Infectious Diseases, School of Public Health, Xiamen University, Xiamen, Fujian, China; n Jinja Publishing Ltd, Bishop’s Stortford, UK; o Immunization, Vaccines and Biologicals Department, World Health Organization, Geneva, Switzerland; p Faculty of Medicine, Universidad Nacional Autónoma de México, Mexico City, Mexico; Chair, Strategic Advisory Group of Experts on Immunization (SAGE)

**Keywords:** Vaccination, Policy, GVAP, Global Vaccine Action Plan, IA2030, Immunization Agenda 2030, Immunization, Global health

## Abstract

The Global Vaccine Action Plan 2011–2020 (GVAP) was developed to realize the ambitions of the Decade of Vaccines – that all individuals and communities enjoy lives free from vaccine-preventable diseases. It included a comprehensive monitoring and evaluation/accountability framework to assess progress towards global targets with recommendations for corrective actions. While many of the GVAP targets are very unlikely to be met by the end of 2020, substantial progress has nevertheless been made, establishing a strong foundation for a successor global immunization strategy, the Immunization Agenda 2030 (IA2030). The Strategic Advisory Group of Experts on immunization has made a series of recommendations to ensure that the lessons learned from GVAP inform the development and implementation of IA2030.

## Highlights

• GVAP provided a comprehensive and coherent global framework for immunization.
• Much progress was achieved under GVAP, although most GVAP goals will not be met.
• GVAP was perceived as top-down, with too little consideration of country context.
• GVAP was only partially implemented, and it had limited levers to influence country actions.
• Many targets were seen as unrealistic, particularly in the absence of additional funding.
• Lessons learned from GVAP can inform the Immunization Agenda 2030.

## 1 Introduction

The Decade of Vaccines Collaboration was launched in 2010 to develop a shared strategy to extend the benefits of vaccination to all. The Collaboration was led by the World Health Organization (WHO), UNICEF, Gavi The Vaccine Alliance, the US National Institute of Allergy and Infectious Diseases (NIAID), and the Bill & Melinda Gates Foundation (BMGF). It was coordinated by a secretariat based at the Instituto de Salud Global Barcelona, Spain, and funded by the BMGF.

The Collaboration assembled working groups to draft the Global Vaccine Action Plan 2011–2020 (GVAP) [1], engaging with stakeholders from more than 140 countries and 290 organizations. An additional working group developed a monitoring, evaluation and accountability (M&E/A) framework.

Ministers of health unanimously endorsed GVAP at the 2012 World Health Assembly (WHA) and took note of the M&E/A framework a year later. In the following years, Regional Vaccine Action Plans and national multi-year plans were developed or updated to align with GVAP.

GVAP drew together immunization goals previously endorsed by the WHA or by WHO Regional Committees and set ambitious new targets in other areas (see Box 1). Pre-existing goals included eradicating polio, eliminating measles and rubella region by region, eliminating maternal and neonatal tetanus from priority countries, and achieving high and equitable vaccination coverage. A range of indicators were developed to monitor progress towards GVAP goals and strategic objectives, including country ownership, financing, service integration, data quality, vaccine availability, and progress in the development and deployment of new vaccines and other innovations.

Box 1 **GVAP at a Glance [1]**

**Vision** A world in which all individuals and communities enjoy lives free from vaccine-preventable diseases.

**Guiding principles.**

**Country ownership** - Countries have primary ownership and responsibility for establishing good governance and for providing effective and quality immunization services for all
**Shared responsibility and partnership** – Immunization against vaccine-preventable diseases is an individual, community and governmental responsibility that transcends borders and sectors
**Equity** – Equitable access to immunization is a core component of the right to health
**Integration** –Strong immunization systems, as part of broader health systems and closely coordinated with other primary health care delivery programmes, are essential for achieving immunization goals
**Sustainability** – Informed decisions and implementation strategies, appropriate levels of financial investment, and improved financial management and oversight are critical to ensuring the sustainability of immunization programmes
**Innovation** – The full potential of immunization can only be realized through learning, continuous improvement and innovation in research and development, as well as innovation and quality improvement across all aspects of immunization

**Goals.**

Achieve a world free of poliomyelitis
Meet vaccination coverage targets in every region, country and community
Exceed the Millennium Development Goal 4 target for reducing child mortality
Meet global and regional elimination targets
Develop and introduce new and improved vaccines and technologies

**Strategic objectives.**

All countries commit to immunization as a priority
Individuals and communities understand the value of vaccines and demand immunization as both their right and responsibility
The benefits of immunization are equitably extended to all people
Strong immunization systems are an integral part of a well-functioning health system
Immunization programmes have sustainable access to predictable funding, quality supply, and innovative technologies
Country, regional, and global research and development innovation maximize the benefits of immunization

To ensure continuity between GVAP and a successor global immunization strategy (the Immunization Agenda 2030, IA2030), a review was undertaken of progress during the Decade of Vaccines to date and of stakeholder perceptions of GVAP and its contributions to this progress [2], [3]. The review was overseen by the Strategic Advisory Group of Experts on Immunization (SAGE) Decade of Vaccines Working Group and endorsed by SAGE in October 2019 [4]. The analysis of stakeholder perceptions has been based on a series of surveys and interviews conducted between 2017 and 2019, details of which have been published elsewhere [2], [3], [5], [6]. In addition to this quantitative and qualitative evidence, this commentary is informed by extensive input from past SAGE Working Group members, GVAP secretariat representatives (from BMGF, Gavi, UNICEF, NIAID, and WHO headquarters and regional offices), as well as civil society organization (CSO) representatives and other immunization stakeholders. The review, took into account all previous published GVAP reports and annexes, in particular the annual SAGE GVAP assessment reports [7] and secretariat reports [8].

### 1.1 Progress during the decade

Most GVAP goals and targets are not likely to be achieved by 2020 ([2], [9]). Nevertheless, steady progress has been made in many areas. All immunization uptake data cited below are from the full SAGE report: Global Vaccine Action Plan review and lessons learned, World Health Organization 2019 [2].

**Fig. 1:**
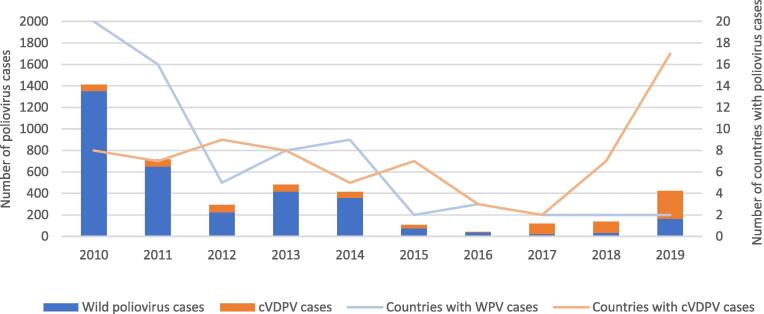
Global wild poliovirus (WPV) and circulating vaccine-derived poliovirus (cVDPV) cases, 2010–2019 Source: WHO, https://extranet.who.int/polis/public/CaseCount.aspx, accessed 30 January 2020.

**Polio eradication**: Wild poliovirus type 2 was certified as eradicated in 2015 and wild poliovirus type 3 in October 2019. Wild poliovirus type 1 currently appears to be circulating only in Afghanistan and Pakistan, where polio eradication efforts face major security, community acceptance challenges and health system fatigue in some instances. Of concern, vaccine-derived poliovirus is increasingly in circulation in multiple countries (Fig. 1).

**Global and regional elimination targets**: Vaccination has reduced the reported incidence of **measles** by 83% since 2000, preventing over 20 million deaths. However, during the past decade, measles outbreaks have occurred in all regions, and global incidence doubled between 2017 and 2018; this trend continued in 2019. All six WHO regions have targeted measles elimination, but global routine infant measles-containing vaccine first dose (MCV1) coverage has plateaued at around 86%, with major variations in coverage across and within countries. Global routine measles-containing-vaccine second dose (MCV2) coverage has increased steadily, from 42% in 2010 to 69% in 2018. However, nearly one-third of all children still do not receive two doses of measles-containing vaccine, and not all countries have yet introduced a second dose. Even in countries with high coverage, pockets of unvaccinated children and adults perpetuate the risk of measles outbreaks.

As of December 2018, 168 out of 194 countries have implemented rubella vaccination. By the end of 2019, four WHO regions have set elimination targets (the Americas, Europe, South-East Asia and the Western Pacific), and the Region of the Americas is rubella-free. Progress towards **maternal and neonatal tetanus** elimination (MNTE) has been steady but the global target – elimination in 40 priority countries – is unlikely to be met by the end of 2020. As of July 2019, only 28 of these countries have validated MNTE.

**Meet vaccination coverage targets in every region, country and community:** Coverage for the third dose of diphtheria, tetanus and pertussis (DTP) in infants has plateaued at about 86% globally across the period from 2010 to 2018 i.e. little change across the decade. Nevertheless, as a result of population growth, more children than ever are receiving three doses of DTP before their first birthday – 116 million infants in 2018, about 5 million more than in 2010. Furthermore, 95 countries sustained 90% DTP3 coverage between 2010 and 2018. Yet, every year, nearly 20 million infants do not receive the full infant course.

**Fig. 2:**
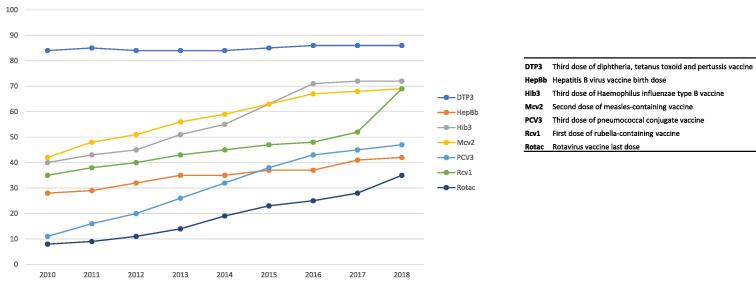
Global coverage for selected vaccines (in percent) for children by 12 months of age, 2010–2018; exception MCV2 at 24 months of age Source: WHO-UNICEF estimates of national immunization coverage 2010 to 2018, July 2019 release. https://www.who.int/immunization/monitoring_surveillance/routine/coverage/WUENIC_notes.pdf?ua=1.

Globally, apart from DTP and BCG (data not shown), coverage has increased for many vaccines (Fig. 2). (Of note, BCG, DTP1 and others are not included in Fig. 2 to make the graph easier to read.) Vaccination coverage rates vary substantially between countries and regions, and while national wealth is an important factor, it is not the only driver of success – some low-income countries have achieved high and equitable coverage while several high-income countries are performing poorly. Global averages are affected by persistent low coverage in several countries over the decade, many affected by conflict, political instability and weak health systems.

**Fig. 3:**
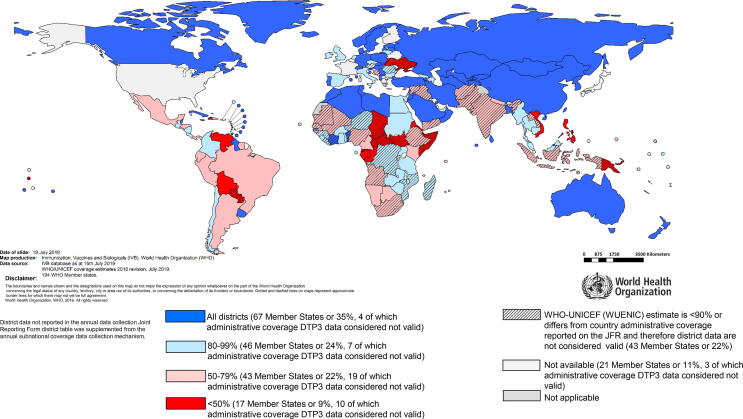
Countries by the percentage of districts with DTP3 coverage >= 80% and valid data, 2018. Source: Joint Reporting Form data, July 2019 release. https://www.who.int/immunization/global_vaccine_action_plan/GVAP_secretariat_report_2019.pdf#page=15. Disclaimer: The boundaries and names shown and the designations used on this map do not imply the expression of any opinion whatsoever on the part of the World Health Organization concerning the legal status of any country, territory, city or area or of its authorities, or concerning the delimitation of its frontiers or boundaries. Dotted lines on maps represent approximate border lines for which there may not yet be full agreement.

While coverage has improved for numerous vaccines, many inequities remain within as well as among countries. Only about one-third of countries meet the target of 80% or greater coverage of the third dose of DTP in every district (Fig. 3).

**Fig. 4:**
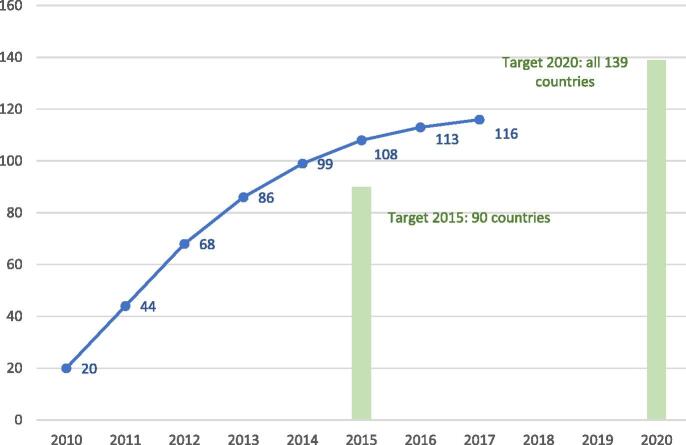
Number of low- and middle-income countries that have introduced and sustained for at least one year, one or more new or underutilized vaccines* between 2010 and 2017 and GVAP targets. *among the following: Hib-containing vaccine, pneumococcal conjugate vaccine, rotavirus vaccine, human qqpapillomavirus vaccine, rubella-containing vaccine and Japanese encephalitis virus vaccine. Source: Joint Reporting Form data, July 2019 release. https://www.who.int/immunization/global_vaccine_action_plan/GVAP_secretariat_report_2019.pdf#page=18.

**Develop and introduce new and improved vaccines and technologies:** Between 2010 and 2017, 116 low- and middle-income countries (LMICs) have introduced and sustained, for at least one year, one or more new vaccines in their programme (Fig. 4). The GVAP target to introduce at least one new vaccine by 2020 in all 139 LMICs will likely be missed but only by a small margin. Non-Gavi-supported middle-income countries have been relatively slow to introduce newer vaccines as they need to self-fund and these vaccines are often costly. Particularly notable over the decade was the widespread introduction of a meningococcal group A vaccine (‘MenAfriVac’), designed specifically for use in Africa. Use of the vaccine has virtually eliminated meningitis A disease in the 26 countries of the African ‘meningitis belt’ [10], [11].

**Fig. 5:**
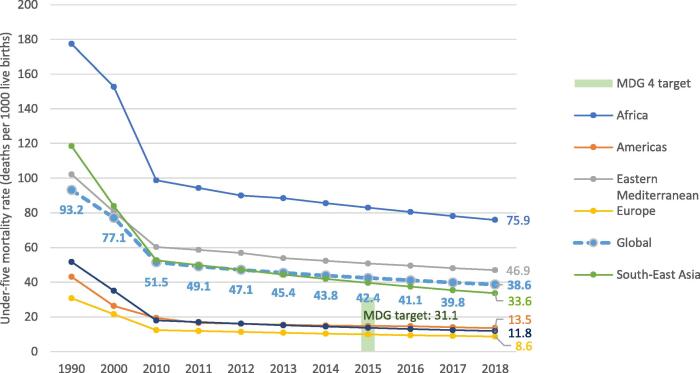
Global and regional under-five mortality rates (per thousand live-births) in 1990, 2000 and from 2010 to 2018. Source: United Nations interagency group for child mortality estimates (UN IGME). 2019 report. https://childmortality.org/wp-content/uploads/2019/10/UN-IGME-Child-Mortality-Report-2019.pdf.

**Exceed the Millennium Development Goal 4 (MDG4) target for reducing child mortality:** Between 2010 and 2018, under-5 mortality declined by 24%, from 52 to 39 deaths per 1000 live births [12] (Fig. 5). The MDG4 target was to reduce the rate of under-five deaths by two-thirds between 1990 (rate 93 per 1000 live births) and 2015 (rate 42 per 1000 live births); with recent progress, this reduction is very close to being achieved. The decrease in vaccine-preventable or partly vaccine-preventable diseases has played a significant part in this mortality reduction [2].

### 1.2 Strategic objectives

**Government expenditure** on national immunization programmes has increased by around one-third in LMICs reporting data. However, government expenditure has shown great year-by-year volatility and has declined in a dozen countries. Many countries have invested in their **evidence-based decision-making capacity**, with the number of countries with National Immunization Technical Advisory Groups (NITAGs) meeting all GVAP process criteria nearly tripling from 40 in 2010 to 114 in 2018 as reported on the WHO UNICEF Joint Reporting Form in 2019.

**Fig. 6:**
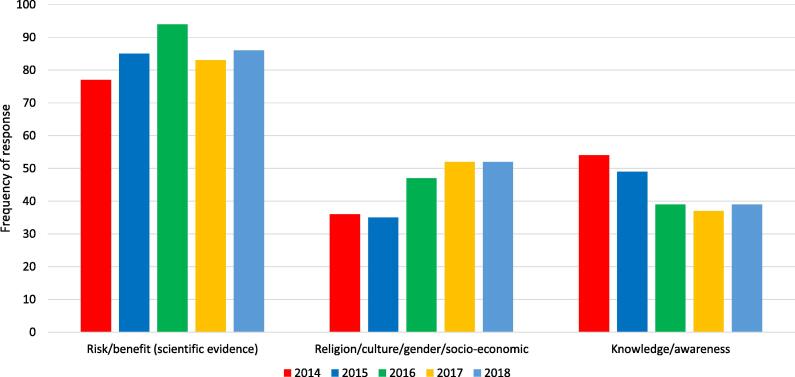
Top three reasons for vaccine hesitancy globally, 2014–2018. Source: Joint Reporting Form data, 2019. https://www.who.int/immunization/global_vaccine_action_plan/GVAP_secretariat_report_2019.pdf#page=15. Note: Data in this chart is reported based on responses to the question “What are the top three reasons for hesitancy in 2018?”. In 2019, only 45% of countries reported to have completed an assessment of hesitancy at national or subnational level in the last five years (2014–2018).

Most countries are now reporting **vaccine hesitancy**, and WHO identified hesitancy as one of its top 10 threats to health in 2019 [13]. Although high-profile anti-vaccination campaigns have received much attention, and have gained exposure through the explosive growth of social media, the causes of hesitancy are complex, spanning awareness of the benefits of vaccination, perceptions of risk of disease, and the quality and convenience of services as well as safety concerns (Fig. 6) [14].

A greater focus on data in GVAP has raised awareness of **subnational inequities** in coverage. Reaching underserved communities – particularly the urban poor, mobile, migrating and displaced populations, and the socially marginalized – remains a major challenge in many countries.

Some **integration of immunization with other health services** has been seen, for example of antenatal care and maternal tetanus immunization, as well as school-based human papillomavirus virus (HPV) vaccination. Immunization continues to have a greater reach than other health services, suggesting there are major opportunities to use immunization to improve access to other services.

Some progress has been made in improving the **affordability** of certain vaccines, particularly through Gavi support. However, many non-Gavi-supported middle-income countries that do not benefit from regional or global procurement mechanisms still report that the cost of vaccines is a major obstacle to their introduction. To help address their needs, WHO launched the Market Information for Access to Vaccines (MI4A) initiative [15] to enhance vaccine-pricing transparency, better link global supply and national needs, and improve countries’ vaccine procurement capacities.

The past decade has seen significant progress in **research and development (R&D)**. During the decade, an improved vaccine for typhoid and novel vaccines for dengue, meningitis B and cholera have been licensed and begun to be used. For the first time, a malaria vaccine is being implemented in routine immunization programmes, through pilot introductions in three African countries, and an Ebola vaccine was successfully deployed in the Ebola outbreaks in West Africa and the Democratic Republic of the Congo.

### 2.1 Lessons learned and reflections

GVAP targets were bold and aspirational, designed to catalyse action and significant progress has been made. However, for some regions and in some areas, the targets and timelines for their achievement may have been unrealistic. Furthermore, many of the ‘failures’ to achieve targets reflect highly challenging circumstances – particularly the impact of conflict, political instability, and displacement, migration, urbanization and vaccine hesitancy, which emerged as major challenges through the decade.

Notably, GVAP provided a comprehensive and coherent strategy, and a global framework for addressing key issues in immunization. It established a common vision and a forum in which immunization stakeholders could collectively discuss matters of concern, as well as a mechanism to connect the activities of global partners. It also acted as a means to focus attention and intention, maintaining the high global profile of immunization. GVAP spanned both disease elimination/eradication initiatives and national immunization programme activities. Importantly, it also included a focus on research and development of new vaccines and vaccine technologies. It provided a solid monitoring, evaluation and accountability framework, which allowed progress to be measured and matters of concern to be raised in a timely manner. This monitoring, evaluation and accountability framework was also implemented at the regional level and in some instances adapted to national and community levels. The structures and relationships established through GVAP provide a sound foundation for the Immunization Agenda 2030.

Furthermore, GVAP’s goals and objectives remain highly relevant today. Increasing coverage, addressing inequities, and eliminating vaccine-preventable infectious diseases remain urgent global priorities. Vaccine security, hesitancy and stimulation of individual and community support for immunization programmes, and the financial sustainability of national immunization programmes are still core challenges. An issue not specifically addressed in GVAP was the need for special mechanisms to ensure access to vaccination services in emergency settings, for displaced people, and for migrants within and passing through countries.

However, there are important lessons to be learned from GVAP and its implementation that can help to shape the Immunization Agenda 2030. Despite extensive consultation, GVAP was widely seen as a **top-down strategy**, focused on global goals and targets. Furthermore, in line with the principle of equity, it aspired to achieve similar goals for all countries, but did not make sufficient allowance for their differing statuses. As a result some regions, such as the African Region, set regional targets that were less ambitious than those in GVAP. In addition, contrary to the expectations of many, GVAP did not come with additional resources. This led to targets and timelines that were perceived by some countries to be unrealistic. As a result, some countries and partners adopted a ‘pick and choose’ approach to GVAP goals, according to their own priorities, rather than fully committing to all aspects of GVAP.

These findings argue for a more **country-focused** approach in the future, with the core of the strategy based on strengthening of national immunization programmes. Strong and sustainable national immunization programmes, embedded in primary health care systems, are an essential foundation for future success. Global and regional partners should provide targeted technical assistance according to the specific needs identified by countries, including supply side management support. **Target setting** also needs to be more sensitive to countries’ current situations and priorities, to provide ambitious but realistically achievable national roadmaps towards globally agreed upon targets.

Global disease--elimination/eradication initiatives align activities around specific goals, energize action and can effectively mobilize resources. However, these specialized and resource-intensive strategies can also draw attention and investment away from broader, long-term programme goals and other vaccine-preventable diseases, create silos and lead to duplication of efforts. As illustrated by polio eradication, they can create infrastructure that benefits immunization programmes more generally and can be a test-bed of innovative new technologies [16], [17]. However, the sustainability of these structures becomes an issue when dedicated funding draws to a close as demonstrated by the threat to the African laboratory surveillance network with the scheduled decrease and ultimate cessation of polio eradication funding.

A key challenge for the future will be to ensure that synergies are achieved between efforts to control specific vaccine-preventable diseases and initiatives to strengthen immunization systems, and vice versa. Strong national immunization programmes will provide a more robust platform for multiple disease-focused programmes, which can themselves help to build immunization programme capacity [18].

A further important challenge will be to incentivize and nurture innovation to solve immunization challenges. GVAP gave a high profile to research and development, and there are clear advantages to further strengthening the linkages between national programmes and the global research and development community to ensure that products meet national needs and that pathways to implementation do not face unnecessary obstacles [19]. Further dialogue is required on economic models that both incentivize new vaccine research and development and ensure availability in LMICs.

In addition, there is scope for innovation in multiple other areas, from vaccine delivery technology (e.g. microarray patches [20]), to digital tools for improving data collection and programme performance assessment [21]. Such tools have the potential to enhance the reach of immunization programmes and improve efficiency, but moving from proof of concept to scale up and use in LMICs often remains challenging [22].

In the absence of convenient indicators, GVAP monitoring paid relatively little attention to operational, implementation and behavioural research. Looking forward, there is continuing need for this kind of research to identify and promote the uptake of innovations that improve the quality of services delivered to communities and optimize programme performance.

An important lesson from GVAP is that, while it recognized essentially all the key issues in immunization and articulated approaches to address them, it was less able to influence the actions of countries to achieve GVAP goals. In addition, coordination across partners was often suboptimal. A future governance model should be based on stronger relationships between core partners and more clearly defined roles and responsibilities, while allowing for a variety of partnership models to ensure that a wider range of collaborations can be established within and beyond the health sector. Immunization has relevance to many Sustainable Development Goals [23] and should be recognized for its potential contributions to areas such as healthy cities, education, responses to climate change, and economic development through the prevention of disease and disability.

Despite increases in both domestic and donor financial support, resourcing has not been sufficient to achieve GVAP targets. Greater awareness of the economic and societal returns of immunization should encourage greater domestic investment. With development assistance budgets under pressure in some donor countries, opportunities to diversify sources of support must be sought, recognizing immunization’s contributions to wider health and development agendas, at global, regional and national levels.

Indeed, the future of immunization needs to be seen in a more integrated context. As marked by the 2018 Astana Declaration [24], the past decade has seen a renewed emphasis on primary health care, as a key route to achieve universal health coverage. Delivering immunization as part of a package of interventions is also likely to be more people-centred. Extending the benefits of vaccination across the life course [25] will also call for collaboration across and beyond health, for example to vaccinate more young people against HPV and older people against influenza.

Furthermore, in many cases, immunization should be but one part of integrated disease control programmes e.g. for cholera, malaria , yellow fever or integrated health care programs e.g. for congenital rubella syndrome, neonatal tetanus,– again calling for strong inter-sectoral collaboration.

Strong and mutually respectful relationships with communities and civil society organizations (CSOs) will also be key to future progress. As access to vaccination improves through supply-side initiatives, coverage will be increasingly dependent on the willingness of individuals to seek out immunization services. CSOs are important partners in relationship building with communities. They are also a diverse constituency, including professional societies and academic institutions as well as community-focused organizations, all of whom could be more actively engaged in many aspects of national immunization programme activities. Additional engagement should also be encouraged with the private health care sector, which plays a key role in immunization in many countries.

Other major trends from the past decade have included the concerted international focus on **global health security** and **antimicrobial resistance**, where again immunization is pivotal to future health. As well as well-immunized populations, vaccine-preventable disease surveillance and the laboratory networks that support surveillance are key elements in global health security. The rapid development of an effective Ebola vaccine and its evaluation in an outbreak setting has been a remarkable success story, and the R&D Blueprint [26] and bodies such as the Coalition for Epidemic Preparedness and Innovations (CEPI) provide examples of infrastructure to accelerate the development of vaccines and diagnostics for emerging and re-emerging infections [27]. Warnings about the severe impact of antimicrobial resistance [28] heighten the need to prevent infections (viral as well as bacterial) to reduce antibiotic usage [29]. Bodies such as the WHO’s Product Development for Vaccines Advisory Committee (PDVAC) can help to ensure that national disease burdens and the needs of LMICs are addressed, as well as global health security, and inform prioritization of vaccine research and development. To facilitate the rapid pace of innovation, WHO should continue to strengthen national regulatory authorities noting the success of regional initiatives such as the African Vaccine Regulatory Forum which accelerated the approval of the rVSV-ZEBOV Ebola vaccine through collective review by the partnering regulatory authorities [30]. Given the pace of development of new vaccines, more work must be done on public receptivity to novel vaccine products and related preventive interventions such as broadly neutralizing antibodies.

GVAP developed comprehensive data-reporting mechanisms from the country up to global level. However, too little of this intelligence influenced activities in countries and at the point of delivery. In the future, a stronger focus is needed on ensuring fit-for-purpose data are available at all levels – from national programmes to individual facilities. At the same time, the capacity to use data to guide action needs to be developed across the immunization workforce. National immunization programmes need to ensure that routine data is seen as a tool to improve performance, and also that new data collection, for example in projects with research partners, can be a way to evaluate innovations to improve reach and efficiency of immunization systems. Immunization data initiatives need to be embedded within or compatible with wider health information systems and not built as siloed tools.

A key related point is that, despite the wealth of data generated by GVAP, the extensive strong monitoring and evaluation/accountability (M&E/A) framework put in place was not sufficient to achieve accountability [6]. A future strategy should ensure a closer link between M&E/A and implementation, so progress towards national targets can be assessed and corrective action taken when necessary.

#### 2.2 Recommendations

Based upon the observations and reflections noted above, the SAGE Working Group made seven high-level recommendations directed to countries, regions and global partners (see Box 2).

Box 2 **SAGE High-level recommendations.**

**A post-2020 global immunization strategy should:**

**1. Build on GVAP’s lessons learned, ensuring more timely and comprehensive implementation at global, regional and national levels.**

**2. Have a key focus on countries:**

2a. Place countries at the centre of strategy development and implementation to ensure context specificity and relevance.

2b. Strengthen country-led evidence-based decision-making.

2c. Encourage the sourcing and sharing of innovations to improve programme performance.

2d. Promote use of research by countries to accelerate uptake of vaccines and vaccine technologies and to improve programme performance.

**3. Maintain the momentum towards GVAP’s goals:**

3a. Incorporate key elements of GVAP, recognizing its comprehensiveness and the need to sustain immunization’s successes each and every year.

3b. Add a specific focus on humanitarian emergencies, displacement and migration, and chronic fragility.

3c. Encourage stronger integration between disease-elimination initiatives and national immunization programmes.

3d. Encourage greater collaboration and integration within and outside the health sector.

**4. Establish a governance model better able to turn strategy into action:**

4a. Create a robust and flexible governance structure and operational model based on closer collaboration between partners.

4b. Incorporate the flexibility to detect and respond to emerging issues.

4c. Develop and maintain a strong communications and advocacy strategy.

**5. Promote long-term planning for the development and implementation of novel vaccine and other preventive innovations, to ensure populations benefit as rapidly as possible.**

**6. Promote use of data to stimulate and guide action and to inform decision-making.**

**7. Strengthen monitoring and evaluation at the national and sub-national level to promote greater accountability.**

More detailed technical recommendations can be found in reference [2], pages 23–27.

## 3 Conclusions

Immunization has saved millions of lives over the past decade, and spared many more from life-changing disability. Looking forward, by some estimates, 122 million premature deaths are likely to be averted by immunization over the lifetime of people born between 2000 and 2030 [31]. Prevention of infectious diseases saves households, especially the poorest, from the crippling social and economic impacts that can occur when a child or adult is sick within a household, with the burden of care falling upon women [32].

These statistics speak to the extraordinary impact of vaccination. They are almost uniquely successful interventions that improve health – highly effective, extremely safe, mostly affordable and hardly affected by the development of antimicrobial resistance.

Indeed, immunization is so successful that it is easy to take them for granted. Recent outbreaks should be warning signs against complacency. Vaccination is above all an investment in the future, delivering a return many times over in averted healthcare costs and enhanced productivity. They are therefore one of the bedrock of future economic and social development.

Over the past 30 years national immunization programmes have achieved astounding success. Now, mainly tough challenges remain – increasing country ownership and sustaining domestic financing, reaching remote rural communities, poor urban populations, mobile and displaced populations, reducing perinatal mortality, ensuring that people actively choose vaccination and have access to convenient and high-quality services across their life span, and developing vaccines against evasive pathogens. Countries need to identify the best ways to solve their unique challenges, sharing their expertise and the experiences of their peer countries. Regions have a critical role to play in translating a global strategy into actions across communities of countries and coordinating support.

Immunization has arguably been ahead of the game in recognizing that infectious disease challenges require global, coordinated solutions. Long-lasting global successes will be achieved through the collective advancement of each and every country supported by effective collaborations. This will be a cornerstone to attain the vision for the next decade of a world where everyone, everywhere, at every age, fully benefits from vaccination for good health and well-being.

## Disclaimer

The authors alone are responsible for the views expressed in this article and they do not necessarily represent the views, decisions or policies of the institutions with which they are affiliated.

## Declaration of Competing Interest

The authors declare that they have no known competing financial interests or personal relationships that could have appeared to influence the work reported in this paper.
